# High-value decisions are made quickly, with no consistent effect on accuracy

**DOI:** 10.1093/pnasnexus/pgaf363

**Published:** 2025-11-13

**Authors:** Angelo Pirrone, Giovanni Sala, Nathan J Evans

**Affiliations:** Department of Psychology, University of Liverpool, Bedford Street South, Liverpool L69 7ZA, United Kingdom; Department of Psychology, University of Liverpool, Bedford Street South, Liverpool L69 7ZA, United Kingdom; Department of Psychology, University of Liverpool, Bedford Street South, Liverpool L69 7ZA, United Kingdom

**Keywords:** decision-making, value-sensitivity, computational models, overall value, collapsing thresholds

## Abstract

High-value decisions tend to be made more quickly. For instance, decision-makers are generally faster when choosing between two preferred options than when choosing between two less preferred options. Several theories have been developed to explain why people are faster for higher overall values, such as facilitation of information processing, reduced caution, or increased processing noise. Importantly, these theories make different predictions for how overall value should influence accuracy, though current results in the literature provide mixed conclusions. Here, we reanalyzed data from 40 previous studies to examine whether decision accuracy is consistently influenced by the overall value of the options. We find that, aside from low-level stimuli-driven effects, decision accuracy does not show a consistent pattern of increase or decrease based on overall value. Our results suggest that earlier claims of a systematic effect of overall value on decision accuracy may have been premature. We provide a mechanistic account of results, discuss why these results may challenge many prevailing theories of decision-making, and highlight open questions for future research.

## Size matters

Previous research has shown that across various tasks and species, decision-makers tend to make faster decisions when the overall value (OV) of alternatives is high compared to when it is low (1); a phenomenon known as value-sensitivity (or magnitude-sensitivity).^a^ For instance, humans make quicker choices when selecting between high-value snack foods (2), or when selecting between perceptual stimuli of high intensity (1, 3, 4); monkeys are faster when presented with options associated with higher average juice concentrations (5), and even aneural unicellular organisms exhibit faster foraging behaviors when food options have higher average quality (6). Crucially, value-sensitivity challenges normative decision-making theories and some well-established computational models, which previously assumed that decisions were driven solely by relative value—the advantage or disadvantage of one option compared to another—irrespective of the OV of the alternatives (1).

While recent research has attempted to understand what cognitive process drives value-sensitivity, developing comprehensive and well-validated theories is difficult without understanding how OV influences decision accuracy, as this relationship has significant implications for understanding the mechanisms behind value-sensitivity. For example, within the evidence accumulation model framework—a dominant framework for explaining how people make decisions (1, 4)—a reduction in response times and an increase in accuracy under high-value conditions would suggest that heightened sensitivity to OV facilitates information processing (2, 7). Conversely, a decrease in response times coupled with a reduction in accuracy could be attributed to reduced caution—with individuals feeling less compelled to carefully evaluate the options when both appear favorable (3, 8)—or increased processing noise under high OV (3, 4).

However, the impact of OV on decision accuracy remains unclear across decision domains (e.g. preferential vs. perceptual choices), and even within specific sub-domains (e.g. food choices). Some early studies documented or predicted, based on theory and modeling work, that accuracy decreases as OV increases (1, 3, 4, 9). More recent work focusing specifically on decision accuracy has found that while response times (RTs) consistently decrease with increasing OV, accuracy actually improves (2, 7). Other studies have reported no significant change in accuracy with varying OV, or have observed an unclear effect (4, 10). Thus, the relationship between OV and decision accuracy remains ambiguous, and this study aims to clarify this relationship to allow for the development of more comprehensive theories of value-sensitivity. By reanalyzing previous studies we show that, with the exception of low-level stimuli-driven effects, decision accuracy does not consistently vary with OV.

## Materials and methods

To examine the effect of OV on decision accuracy, we reanalyzed existing two-alternative forced-choice studies. Studies were included based on several criteria. First, the OV manipulation had to vary across at least two levels (e.g. low OV vs. high OV). Second, studies needed to employ a free-response protocol, allowing participants to respond in their own time. Third, raw trial-level data, including accuracy for each trial, had to be available. Since the practice of sharing raw data is relatively recent, most “older” studies did not meet this criterion. For multichoice tasks (e.g. initial choice, confidence rating, second choice), we focused solely on data from the initial choice task. Studies with complex designs, such as those involving resource allocation between self and others, were excluded. Finally, we decided to focus exclusively on human decision-making.

To be included in the analyses, studies had to use stimuli that fit into one of four categories: brightness discrimination, numerical value tasks, preference choice, and learned-value association tasks. Brightness discrimination tasks required participants to select the brighter stimulus (3, 5, 6, 11). Numerical value tasks involved choosing between numerical values or gambles with varying probabilities and payoffs (12–14). Preference choice tasks involved selecting preferred items, often food but also abstract images or posters (2, 14, 15). Learned-value association tasks involved pairing abstract stimuli with values such as points or money (2, 16), followed by binary choices.^b^

To identify relevant studies, we followed a multistep approach. We began with articles explicitly focused on value-sensitivity, cited in the relatively limited literature on the topic (1–4, 7, 17), many of which had or referenced publicly available data that met our criteria. Next, we solicited datasets by emailing the Cognitive Science Society and Judgment and Decision Making mailing lists. Finally, to reduce publication bias regarding value-sensitivity, we searched OSF and Google Scholar for raw data meeting our inclusion criteria. This search identified 12 eligible datasets. These studies did not specifically focus on OV effects, minimizing selection bias related to OV’s impact on decision-making. We set a stopping criterion of 40 datasets.

As summarized in Table 1, the studies varied by domain (perceptual vs. preferential) and stimulus type (brightness, numbers, preference, and abstract).^c^

**Table 1.** Summary of reanalyzed studies.

ID	Study	Domain	Stimuli	Nppt	Ntrials	$b_{rt}$	${SE}_{b_{rt}}$	$p_{b_{rt}}$	$b_{acc}$	${SE}_{b_{acc}}$	$p_{b_{acc}}$	$b_{{acc}_{Bayes}}$	${BF}_{acc}$
1	Hare et al. (22)	Preferential	Abstract	19	570	− 0.022	0.014	0.112	− 0.199	0.133	0.135	− 0.169	Anecdotal evidence for H0
2	Pirrone et al. (21)	Preferential	Abstract	21	42,826	− 0.025	0.008	0.007	− 0.092	0.049	0.062	− 0.096	Anecdotal evidence for H0
3	Konovalov and Krajbich (20)	Preferential	Abstract	45	5,609	− 0.07	0.012	<0.001	0.03	0.064	0.638	0.026	Moderate evidence for H0
4	Shevlin et al. (16)	Preferential	Abstract	52	14,383	− 0.077	0.007	<0.001	0.068	0.026	0.009	0.067	Anecdotal evidence for H1
5	Shevlin et al. (2)	Preferential	Abstract	70	18,406	− 0.039	0.005	<-.001	0.149	0.05	0.003	0.136	Moderate evidence for H1
6	Pirrone et al. (5)	Perceptual	Brightness	9	12,600	− 0.008	0.011	0.5	− 0.497	0.051	< .001	− 0.507	Extreme evidence for H1
7	Ko et al. (23)	Perceptual	Brightness	35	33,755	− 0.015	0.005	0.003	− 0.325	0.029	< .001	− 0.328	Extreme evidence for H1
8	Teodorescu et al. (3)	Perceptual	Brightness	7	8,400	− 0.037	0.011	0.009	− 0.305	0.063	<0.001	− 0.307	Strong evidence for H1
9	Teodorescu et al. (3)	Perceptual	Brightness	7	8,398	− 0.034	0.005	<0.001	− 0.269	0.035	<0.001	− 0.263	Very strong evidence for H1
10	Ko et al. (23)	Perceptual	brightness	37	35,562	− 0.008	0.004	0.082	− 0.252	0.028	< .001	− 0.254	Extreme evidence for H1
11	Turner et al. (24)	Perceptual	Brightness	30	25,931	− 0.01	0.004	0.007	− 0.23	0.028	<0.001	− 0.234	Extreme evidence for H1
12	Ting and Gluth (7)	Perceptual	Brightness	61	11,514	− 0.053	0.007	< 0.001	0.056	0.028	0.044	0.045	Moderate evidence for H0
13	Edmunds et al. (14)	Preferential	Numbers	56	2,232	0.001	0.011	0.922	− 0.149	0.052	0.004	− 0.169	Strong evidence for H1
14	Shevlin et al. (2)	Preferential	Numbers	75	19,198	0.015	0.009	0.099	− 0.13	0.036	< .001	− 0.139	Very strong evidence for H1
15	Glickman et al. (13)	Perceptual	Numbers	27	13,361	− 0.035	0.004	<0.001	− 0.024	0.03	0.427	− 0.018	Moderate evidence for H0
16	Glickman et al. (13)	Perceptual	Numbers	30	13,401	− 0.013	0.003	<0.001	− 0.012	0.024	0.607	− 0.012	Strong evidence for H0
17	Pirrone (25)	Preferential	Numbers	62	27,900	0.013	0.007	0.057	0.029	0.034	0.398	0.028	Moderate evidence for H0
18	Lee et al. (26)	Perceptual	Numbers	44	8,662	− 0.03	0.007	< .001	0.036	0.031	0.25	0.039	Moderate evidence for H0
19	Edmunds et al. (14)	Preferential	Numbers	54	1,753	0.008	0.008	0.315	0.12	0.055	0.028	0.118	Anecdotal evidence for H1
20	Oud et al. (27)	Preferential	Preference	49	8,955	− 0.059	0.01	< .001	− 0.296	0.151	0.05	− 0.286	Anecdotal evidence for H1
21	Sepulveda et al. (28)	Preferential	Preference	31	3,720	0.047	0.015	0.005	− 0.283	0.058	<0.001	− 0.250	Extreme evidence for H1
22	Folke et al. (29)	Preferential	Preference	28	6,720	− 0.001	0.025	0.982	− 0.281	0.119	0.018	− 0.227	Anecdotal evidence for H1
23	Eum et al. (30)	Preferential	Preference	50	19,000	− 0.041	0.005	<0.001	− 0.048	0.041	0.247	− 0.045	Moderate evidence for H0
24	Smith and Krajbich (31)	Preferential	Preference	42	4,013	− 0.029	0.009	0.004	− 0.025	0.048	0.609	− 0.022	Moderate evidence for H0
25	Pirrone (32)	Preferential	Preference	66	13,200	− 0.056	0.008	<0.001	− 0.0001	0.038	0.997	0.009	Moderate evidence for H0
26	Smith and Krajbich (33)	Preferential	Preference	44	6,453	− 0.071	0.01	<0.001	0.017	0.046	0.704	0.023	Moderate evidence for H0
27	Smith and Krajbich (33)	Preferential	Preference	36	7,200	− 0.035	0.011	0.004	0.02	0.047	0.666	0.021	Moderate evidence for H0
28	Brus et al. (34)	Preferential	Preference	33	5,016	− 0.052	0.016	0.002	0.026	0.047	0.584	0.028	Moderate evidence for H0
29	Smith et al. (35)	Preferential	Preference	27	2,850	− 0.12	0.014	<0.001	0.042	0.074	0.565	0.060	Moderate evidence for H0
30	Smith and Krajbich (31)	Preferential	Preference	42	3,995	− 0.036	0.01	<0.001	0.051	0.05	0.313	0.053	Moderate evidence for H0
31	Krajbich et al. (15)	Preferential	Preference	39	3,647	− 0.061	0.012	<0.001	0.057	0.054	0.294	0.061	Moderate evidence for H0
32	Shevlin et al. (2)	Preferential	Preference	44	10,577	− 0.034	0.008	<0.001	0.063	0.034	0.066	0.067	Anecdotal evidence for H0
33	Ting and Gluth (7)	Preferential	Preference	61	11,526	− 0.068	0.01	<0.001	0.069	0.024	0.004	0.071	Moderate evidence for H1
34	Lee and Daunizeau (12)	Preferential	Preference	41	3,034	− 0.088	0.012	<0.001	0.079	0.058	0.178	0.081	Anecdotal evidence for H0
35	Edmunds et al. (14)	Preferential	Preference	41	3,698	− 0.067	0.012	< .001	0.088	0.072	0.223	0.066	Moderate evidence for H0
36	Chen and Krajbich (36)	Preferential	Preference	44	8,453	− 0.053	0.007	<0.001	0.107	0.047	0.023	0.112	Anecdotal evidence for H1
37	Edmunds et al. (14)	Preferential	Preference	52	2,599	− 0.029	0.01	0.004	0.116	0.065	0.075	0.129	Anecdotal evidence for H0
38	Smith and Krajbich (33)	Peferential	Preference	44	7,236	− 0.044	0.008	<0.001	0.127	0.047	0.007	0.126	Anecdotal evidence for H1
39	Gwinn (37)	Preferential	Preference	36	6,237	− 0.03	0.012	0.02	0.156	0.073	0.033	0.161	Anecdotal evidence for H1
40	Shevlin et al. (2)	Preferential	Preference	50	12,931	− 0.027	0.006	<0.001	0.21	0.046	<0.001	0.211	Extreme evidence for H1

**Fig. 1.**
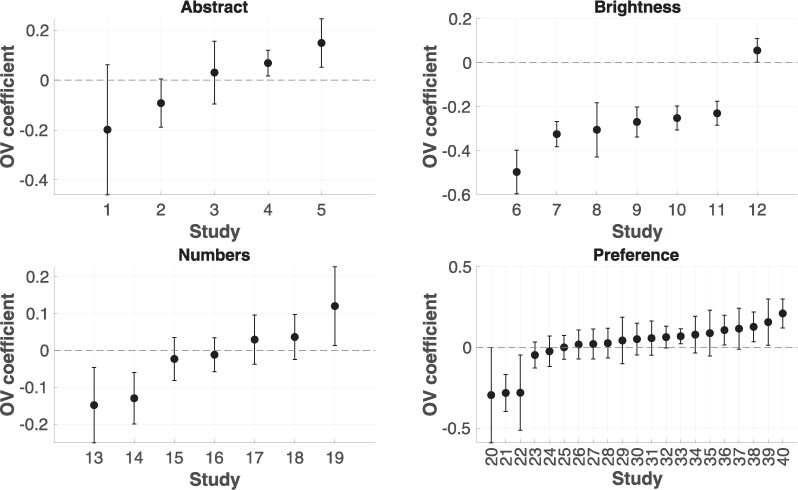
The effect of OV on accuracy. We grouped studies according to the stimulus type. Separately for each study, we performed a mixed-effect logistic regression for the effect of OV and relative value on accuracy. For the ID of each study, refer to Table 1. Bars are 95% CI for the regression coefficient of OV on accuracy.

For each study, we report the ID (corresponding to the *x*-axis in Fig. 1), bibliographic reference (study), domain (perceptual or preferential), stimulus type (brightness, numbers, preference, or abstract), number of participants (Nppt), number of trials (Ntrials), coefficients ($b_{\mathrm{rt}}$, $b_{\mathrm{acc}}$) for OV effects on RTs and accuracy, their standard errors (${\mathrm{SE}}_{b_{\mathrm{rt}}}$, ${\mathrm{SE}}_{b_{\mathrm{acc}}}$), and corresponding *P*-values ($p_{b_{\mathrm{rt}}}$, $p_{b_{\mathrm{acc}}}$). Given that frequentist results pointed to a null effect of OV on accuracy for many studies, we decided to quantify for each study the support for the null hypothesis using Bayesian Regressions with mixed effects. We computed Bayes Factors (BFs) comparing the models in which there is an effect of OV (H1) and model without the effect of OV (H0). H0 was the model with only the effect of difference; for tasks in which difference was kept constant across trials (24, 37), H0 was the intercept-only model. H0 and H1 had the same random-effects structure, so the BFs reflected the support for the fixed effect of OV. For each study we report the coefficient for the effect of OV (standardized) on accuracy, while accounting for the effect of difference ($b_{{\mathrm{acc}}_{\mathrm{Bayes}}}$) and whether analyses showed support for the null hypothesis, or for an effect of OV on accuracy (${\mathrm{BF}}_{\mathrm{acc}}$). For the interpretation of BFs, we relied on classical guidelines (38).

## Analyses

We examined how OV influences response times and accuracy across multiple studies, while controlling for choice difficulty (i.e. the effect of relative value). Linear mixed-effects models were fitted separately for each study to estimate the beta coefficients and their standard errors. These beta coefficients were then incorporated into a meta-analysis for each outcome measure (i.e. response time and accuracy), with each coefficient weighted by the inverse of its squared standard error. The meta-analyses assessed both the overall effect of OV across all studies and the effect of OV by stimulus type (i.e. numbers, preferences, brightness, and abstract stimuli).

For each study, the predictors were OV (the sum of the values of the stimuli, z-scored within each study) and RV (the absolute relative value between the values of the stimuli, z-scored). For each study, log-transformed response times were regressed on standardized OV and RV, with random intercepts and slopes by participant. Similarly, accuracy was regressed on standardized OV and RV, with random intercepts and slopes by participant. See Table 1 for the coefficients and associated *P*-values for the effect of OV on accuracy and RTs (while accounting for RV) for each study. Figure 1 shows, for each study, the effect of OV on accuracy, which is our primary focus.

The meta-analytic model for RTs showed a significant negative overall effect of OV, indicating that higher OV was associated with faster RTs (b=−0.035, SE=0.005, P<0.001). Significant between-study heterogeneity was observed (τ=0.028, P<0.001), suggesting the potential impact of stimulus type on the effect sizes. The overall effect size for OV on RT was robust for abstract (b=−0.047, SE=0.011, P<0.001), preference (b=−0.046, SE=0.06, P<0.001), and brightness stimuli (b=−0.024, SE=0.009, P=0.012), and virtually null for numbers stimuli (b=−0.006, SE=0.009, P=0.495). When equal alternatives only were tested, for datasets with more than 50 equal alternatives trials, we replicated the classical result of response times decreasing as a function of OV (b=−0.042, SE=0.007, P<0.001); since only about half of datasets had equal alternatives, we did not perform further analyses by stimulus type.

The meta-analytic model for accuracy yielded no significant effect of OV on accuracy across all studies (b=−0.038, SE=0.026, P=0.146). Significant between-study heterogeneity was observed (τ=0.154, P<0.001). As shown in Fig. 1, only for brightness stimuli lower accuracy was linked to higher OV: (b=−0.255, SE=0.044, P<0.001). For all other stimulus types, no effect of OV on accuracy was observed (abstract stimuli: b=0.014, SE=0.057, P=0.805, numbers stimuli: b=−0.019, SE=0.044, P=0.67, preference stimuli: b=0.029, SE=0.027, P=0.289). Bayesian analyses (see Table 1) confirmed the results of the frequentist analyses: no consistent effect of OV on accuracy other than for brightness discrimination tasks.

Finally, moderator analysis showed no significant difference between datasets cited in the value-sensitivity literature and those that were not, in either the RT model or the accuracy model (both Ps>0.552).

## Discussion

Our results confirm that the overall value (OV) of alternatives influences response times (RTs) across decision-making tasks, even for equal alternatives. Specifically, decisions for high OV trials are made more quickly than for low OV trials—a well-established pattern in the decision-making literature (1). With regard to the effect of OV on accuracy, which is our primary focus, our results indicate that OV had no significant overall impact on accuracy, except in brightness discrimination tasks where high OV was associated with lower accuracy. To our knowledge, this is the first study that systematically examines the effect of OV on accuracy across tasks and domains. These findings are significant for the development of theories of value-sensitivity, as existing models have proposed that the effect of OV on accuracy should be related to its effect on RTs, either positively (7, 10) or negatively (1, 3, 4, 11).

Several functional or mechanistic explanations can account for an *exclusive* increase or decrease in accuracy as a function of OV (1, 10). For example, in brightness discrimination, stimulus-specific input-dependent noise that scales with OV or response competition between evidence accumulators produced by lateral inhibition have been shown to provide a good quantitative fit to experimental data (3, 4). However, if we accept the widely held view that simple, rapid decisions rely on a shared computational framework across tasks and species, the real challenge is not explaining an exclusive increase or decrease in accuracy, but rather accounting for why both patterns can emerge across different contexts, especially given that RTs consistently decrease as OV increases. One possibility is that although increased OV consistently speeds decision times, the direction of accuracy change depends on how OV modulates other parameters, resulting in context-dependent behavioral outcomes.

**Fig. 2.**
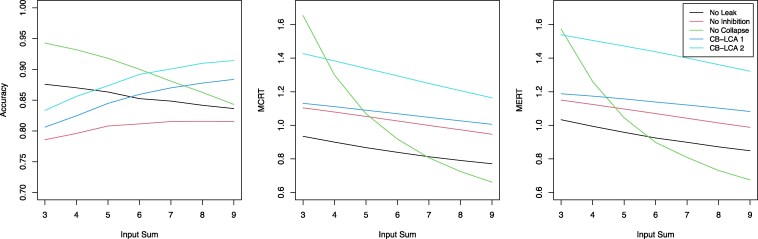
Simulations of different LCA variants to display how accuracy (left panel), mean correct response time (MCRT; middle panel) and mean error response time (MERT; right panel) change over different overall amounts of input (i.e. overall value; *x*-axis), using an efficient method and framework (18). In all cases, the input sum was determined by the sum of the drift rates of two accumulators, with the difference between accumulators always being 2 (e.g. for the input sum of 4, the correct accumulator has a drift rate of 3, and the error accumulator has a drift rate of 1). For all models, the evidence state was truncated at 0, the stochastic noise was fixed at 1, and the nondecision time was fixed at 0.3. The models differed in their lateral inhibition (default of 4, unless stated otherwise), leakage (default of 4, unless stated otherwise), initial threshold (default of 4, unless stated otherwise), and threshold collapse rate (default of 4, unless stated otherwise). For the “no-leak” model (black line), leakage was set to 0. For the “no-inhibition” model (red line), inhibition was set to 0. For the “no collapse” model, the initial threshold was set to 1 and the collapse rate was set to 0. For the “CB-LCA 1” model, all parameters were set to default. For the “CB-LCA 2” model, the initial threshold was set to 3 and the collapse rate was set to 2.

In an attempt to illustrate examples of a unifying computational framework, our simulation analyses (see Fig. 2 and simulation code available on OSF) show that the Leaky Competing Accumulator (LCA) model (19), a computational model of decision-making, generally predicts a decline in accuracy as OV increases, across a wide range of parameter values.^d^ In the LCA, each choice option is represented by a separate evidence accumulator, but this process is shaped by two key mechanisms: leak, which causes a gradual loss of accumulated evidence, and lateral inhibition, where accumulators inhibit each other’s activity, so options with less evidence are suppressed and the one with the most evidence is more likely to be chosen. Crucially, our simulations show that when both leak and inhibition are combined with collapsing decision thresholds, so that the amount of evidence required to make a decision decreases over time, a new pattern emerges: RTs decrease as OV increases, but accuracy improves with higher OV.^e^ Importantly, in these simulations, the rate of threshold collapse over time^f^ is held constant across OV, meaning that the increase in accuracy is purely explained by the dynamics of the model.^g^

Although these findings are preliminary, they suggest that the collapsing thresholds LCA may provide a mechanistic account for the observed variability in OV effects on accuracy. Specifically, the model can accommodate scenarios in which accuracy increases or decreases with OV, while RTs decrease. A promising direction for future research is to examine how differences in experimental design—such as reward structure, static vs. dynamic stimuli, and response time constraints—and factors that differ between individuals—such as attention, motivation, and learning—may interact to modulate changes in accuracy across OV. Importantly, as different LCA parameters modulate how accuracy changes across OV, these different factors may also map onto modulation in specific decision parameters, such as lateral inhibition, leakage, and boundary collapse rate, and potentially explain the diverse and sometimes contradictory effects of OV on accuracy observed across studies.

One key implication of our findings is that previous conclusions regarding value-sensitivity may need to be revised. For example, our results raise questions about the claim that “high-value decisions are fast and accurate,” and that improved information processing alone can explain behavioral changes under increased OV (2). Similarly, it appears unlikely that value-sensitivity can be fully explained by a decision mechanism that speeds up decision-making at the expense of accuracy, as previously suggested by several accounts (for a review, see Ref. 1).

Note that several of the studies reported, such as some of those in the “abstract” category, did not focus directly on OV effects, hence those studies did not control for distortions of difficulty at different levels of OV (1). We welcome more studies that use objective values and unconfounded designs to estimate the effect of OV on accuracy in the preference domain. However, it is still possible to test OV independently of distortions of difficulty by using equal-alternative trials. In such cases, decreases in RTs suggest a mechanism that speeds responses without increasing accuracy, since accuracy-enhancing mechanisms (e.g. increased drift) would be inoperative and expected to yield no change or slower RTs for high OV. In all “abstract” studies (that had equal alternatives), regardless of whether they controlled for distortions of difficulty at different levels of OV (16, 20, 21) we consistently observe a significant RT decreases as a function of OV. These findings are hard to explain with the “enhanced high-value sensitivity” hypothesis alone (2, 16).

Our findings highlight important difference between perceptual and preferential choice, where in the former the effect of OV on accuracy seems more consistently negative (in specific sub-domains), while in the latter the effect is variable. Furthermore, the findings highlight the importance of developing more nuanced theoretical frameworks that can accommodate the diversity of effects observed across different tasks, rather than relying solely on models that best fit individual datasets.

## Data Availability

Analyses scripts are available at osf.io/4v7n6/.

## Notes

a We acknowledge that the term “value” can be ambiguous, as it may be interpreted as economic value (which is often the case in decision neuroscience), subjective value, or even personal beliefs. In our work, “value” is a general concept that refers to stimulus intensity (i.e. the input “values” that feed into the decision process). That is, values are the representations of stimulus intensity—regardless of the nature of the stimuli—that are used in the decision-making process. For instance, this could mean economic value in an economic task, input brightness in a brightness discrimination task, or signal strength in a motion discrimination task.

b In preference tasks, participants’ preferences (i.e. their subjective values) are measured through self-reports prior to making binary choices. In contrast, in learned-value association tasks, the values of the stimuli are assigned by the experimenter. This distinction between preference and learned-value tasks can be seen as broadly reflecting the difference between subjective and “objective” value.

c We will establish a procedure on OSF for future dataset updates, enabling broader investigations into decision-making as the dataset expands.

d Note that we assessed this on a broader range of parameter values in separate simulations, though chose a smaller subset for brevity in the current figure.

e Note that accuracy also slightly increases without lateral inhibition, though all three components combined are necessary to produce larger increases in accuracy.

f It should also be noted that all other parameters were also held constant, with the drift rate for each accumulator being equal to the actual value for that response option.

g Note that the decrease in RT and increase in accuracy is likely caused by the combination of two key dynamics: (i) higher overall drift rates for each accumulator (with same difference in drift rate between accumulators) reaching the thresholds faster in higher OV and (ii) collapsing thresholds causing trials with slower accumulation (in lower OV) to have lower accuracy as less evidence is needed to make a decision with increasing decision time.

## References

[1] Pirrone A, Reina A, Stafford T, Marshall JA, Gobet F. 2022. Magnitude-sensitivity: rethinking decision-making. Trends Cogn Sci. 26(1):66–80. 34750080 10.1016/j.tics.2021.10.006

[2] Shevlin BR, Smith SM, Hausfeld J, Krajbich I. 2022. High-value decisions are fast and accurate, inconsistent with diminishing value sensitivity. Proc Natl Acad Sci U S A. 119(6):e2101508119. 35105801 10.1073/pnas.2101508119 PMC8832986

[3] Teodorescu AR, Moran R, Usher M. 2016. Absolutely relative or relatively absolute: violations of value invariance in human decision making. Psychon Bull Rev. 23(1):22–38. 26022836 10.3758/s13423-015-0858-8

[4] Ratcliff R, Voskuilen C, Teodorescu A. 2018. Modeling 2-alternative forced-choice tasks: accounting for both magnitude and difference effects. Cogn Psychol. 103(4):1–22. 29501775 10.1016/j.cogpsych.2018.02.002 PMC5911219

[5] Pirrone A, Azab H, Hayden BY, Stafford T, Marshall JA. 2018. Evidence for the speed-value trade-off: human and monkey decision making is magnitude sensitive. Decision. 5(2):129–142. 29682592 10.1037/dec0000075 PMC5908478

[6] Marshall JA, Reina A, Hay C, Dussutour A, Pirrone A. 2022. Magnitude-sensitive reaction times reveal non-linear time costs in multi-alternative decision-making. PLoS Comput Biol. 18(10):e1010523. 36191032 10.1371/journal.pcbi.1010523 PMC9560628

[7] Ting C-C, Gluth S. 2025. High overall values mitigate gaze-related effects in perceptual and preferential choices. J Exp Psychol Gen. 154(5):1320–1333. 39899026 10.1037/xge0001723

[8] Pirrone A, Gobet F. 2021. Is attentional discounting in value-based decision making magnitude sensitive? J Cogn Psychol. 33(3):327–336.

[9] Pais D, et al 2013. A mechanism for value-sensitive decision-making. PLoS One. 8(9):e73216. 24023835 10.1371/journal.pone.0073216 PMC3759446

[10] Shevlin BR, Krajbich I. 2021. Attention as a source of variability in decision-making: accounting for overall-value effects with diffusion models. J Math Psychol. 105(4):102594.

[11] Kirkpatrick RP, Turner BM, Sederberg PB. 2021. Equal evidence perceptual tasks suggest a key role for interactive competition in decision-making. Psychol Rev. 128(6):1051–1087. 34014711 10.1037/rev0000284

[12] Lee DG, Daunizeau J. 2021. Trading mental effort for confidence in the metacognitive control of value-based decision-making. Elife. 10:e63282. 33900198 10.7554/eLife.63282 PMC8128438

[13] Glickman M, Moran R, Usher M. 2022. Evidence integration and decision confidence are modulated by stimulus consistency. Nat Hum Behav. 6(7):988–999. 35379981 10.1038/s41562-022-01318-6

[14] Edmunds C, Bose D, Camerer C, Mullett TL, Stewart N. 2020. Accumulation is late and brief in preferential choice [preprint]. PsyArXiv. 10.31234/osf.io/sa4zr

[15] Krajbich I, Armel C, Rangel A. 2010. Visual fixations and the computation and comparison of value in simple choice. Nat Neurosci. 13(10):1292–1298. 20835253 10.1038/nn.2635

[16] Shevlin BR, Smith SM, Hausfeld J, Krajbich I. 2022. Reply to Pirrone and Tsetsos: Robust evidence for enhanced high-value sensitivity. Proc Natl Acad Sci U S A. 119(36):e2209521119. 35969800 10.1073/pnas.2209521119 PMC9459315

[17] Mormann M, Russo JE. 2021. Does attention increase the value of choice alternatives? Trends Cogn Sci. 25(4):305–315. 33549495 10.1016/j.tics.2021.01.004

[18] Evans NJ . 2019. A method, framework, and tutorial for efficiently simulating models of decision-making. Behav Res Methods. 51(5):2390–2404. 30924105 10.3758/s13428-019-01219-z PMC6797646

[19] Usher M, McClelland JL. 2001. The time course of perceptual choice: the leaky, competing accumulator model. Psychol Rev. 108(3):550–592. 11488378 10.1037/0033-295x.108.3.550

[20] Konovalov A, Krajbich I. 2016. Gaze data reveal distinct choice processes underlying model-based and model-free reinforcement learning. Nat Commun. 7(1):12438. 27511383 10.1038/ncomms12438 PMC4987535

[21] Pirrone A, Wen W, Li S. 2018. Single-trial dynamics explain magnitude sensitive decision making. BMC Neurosci. 19(1):1–10. 30200889 10.1186/s12868-018-0457-5 PMC6131863

[22] Hare TA, Schultz W, Camerer CF, O’Doherty JP, Rangel A. 2011. Transformation of stimulus value signals into motor commands during simple choice. Proc Natl Acad Sci U S A. 108(44):18120–18125. 22006321 10.1073/pnas.1109322108 PMC3207676

[23] Ko YH, et al 2022. Divergent effects of absolute evidence magnitude on decision accuracy and confidence in perceptual judgements. Cognition. 225(1):105125. 35483160 10.1016/j.cognition.2022.105125

[24] Turner W, Feuerriegel D, Andrejević M, Hester R, Bode S. 2021. Perceptual change-of-mind decisions are sensitive to absolute evidence magnitude. Cogn Psychol. 124(8):101358. 33290988 10.1016/j.cogpsych.2020.101358

[25] Pirrone A . 2024. Subjective utility modulates the effect of overall stimulus intensity on decision-making. Unpublished Manuscript. https://osf.io/xvnjd/

[26] Lee DG, Tsetsos K, Pezzulo G, Shahar N, Usher M. 2025. Variability and accessibility of information guide gaze dynamics in decision making. Decision. 12(2):146–164.

[27] Oud B, et al 2016. Irrational time allocation in decision-making. Proc R Soc Lond B Biol Sci. 283(1822):20151439. 10.1098/rspb.2015.1439 PMC4721081 26763695

[28] Sepulveda P, et al 2020. Visual attention modulates the integration of goal-relevant evidence and not value. Elife. 9:e60705. 33200982 10.7554/eLife.60705 PMC7723413

[29] Folke T, Jacobsen C, Fleming SM, De Martino B. 2016. Explicit representation of confidence informs future value-based decisions. Nat Hum Behav. 1(1):0002.

[30] Eum B, Dolbier S, Rangel A. 2023. Peripheral visual information halves attentional choice biases. Psychol Sci. 34(9):984–998. 37470671 10.1177/09567976231184878

[31] Smith SM, Krajbich I. 2021. Mental representations distinguish value-based decisions from perceptual decisions. Psychon Bull Rev. 28(4):1413–1422. 33821461 10.3758/s13423-021-01911-2

[32] Pirrone A . 2024. Clarifying the role of processing noise in value-based decision-making. Unpublished Manuscript. https://osf.io/c84z2/

[33] Smith SM, Krajbich I. 2018. Attention and choice across domains. J Exp Psychol Gen. 147(12):1810–1826. 30247061 10.1037/xge0000482

[34] Brus J, Aebersold H, Grueschow M, Polania R. 2021. Sources of confidence in value-based choice. Nat Commun. 12(1):7337. 34921144 10.1038/s41467-021-27618-5 PMC8683513

[35] Smith A, Bernheim BD, Camerer CF, Rangel A. 2014. Neural activity reveals preferences without choices. Am Econ J Microecon. 6(2):1–36. 25729468 10.1257/mic.6.2.1 PMC4339868

[36] Chen WJ, Krajbich I. 2016. Pupil dilation and attention in value-based choice. Unpublished Manuscript (The Ohio State University). Appeared in Smith & Krajbich (2019). https://osf.io/ktsye

[37] Gwinn RE . 2016. Attitudes and attention: how attitude accessibility and certainty influence attention and subjective choice [master’s thesis]. The Ohio State University (Retrieved from osf.io/ktsye).

[38] Jeffreys H . The theory of probability. OUP Oxford, 1998.

